# Genome Sequence of the Enterohemorrhagic *Escherichia coli* Bacteriophage UFV-AREG1 

**DOI:** 10.1128/genomeA.00412-16

**Published:** 2016-10-13

**Authors:** Maryoris E. Soto Lopez, Laís Silva Batalha, Pedro Marcus Pereira Vidigal, Luiz Augusto A. Albino, Delaine Meireles Gouveia Boggione, Marco Tulio Pardini Gontijo, Denise M. Soares Bazzolli, Regina C. Santos Mendonca

**Affiliations:** a Departamento de Tecnologia de Alimentos (DTA), Universidade Federal de Viçosa, Minas Gerais, Brazil; b Núcleo de Análise de Biomoléculas (NuBioMol), Centro de Ciências Biológicas (CCB), Universidade Federal de Viçosa, Minas Gerais, Brazil; c Departamento de Microbiologia (DMB), Universidade Federal de Viçosa, Minas Gerais, Brazil

## Abstract

Here, we present the genome sequence of the *Escherichia coli* bacteriophage UFV-AREG1. This phage was isolated from cowshed wastewater and showed specificity for enterohemorrhagic *E. coli* O157:H7 (ATCC 43895), *E. coli* 0111 (CDC O11ab) and *E. coli* (ATCC 23229).

## GENOME ANNOUNCEMENT

In general, bacteriophages show great diversity, morphology, biochemistry, and genetic content (1). The study of the T4-like bacteriophage genomic sequences has provided countless contributions for these fields, and specially to functional genomics and proteomics (2, 3).

The bacteriophage UFV-AREG1 was isolated from cowshed wastewater showing specificity to *Escherichia coli* O157:H7 (CDC EDL-933), *E. coli* 0111 (CDC O11ab), and *E. coli* (ATCC 23229). Bacteriophage UFV-AREG1 can reach concentrations of over 10^12^ PFU/ml in laboratory conditions when the host is *E. coli* O157:H7. Transmission electron microscopy showed a phage with an icosahedral head of 114 by 86 nm and a contractile tail of 117 by 23 nm. These parameters allow the classification of UFV-AREG1 as a member of the *Myoviridae* family (4, 5). UFV-AREG1 can remain viable after simulation of sanitization processes with sodium dichloroisocyanurate (219 ± 13 mg/l) and hydrogen peroxide (58.1 ± 5 mg/l). On the other hand, this phage lost viability after contact with peracetic acid (91 ± 17 mg/l).

The genome of UFV-AREG1 was sequenced using Illumina HiSeq by Macrogen (Seoul, Republic of Korea), yielding a total of 10,319,880 paired-end reads 100 bp in length. The sequenced reads were trimmed for quality (minimum Q30 score), and the genome sequence was assembled with a coverage of 5,600× using Geneious version 9.1.2 (Biomatters) with the genome of *Escherichia* phage HY01 (KF925357) as the reference. Genes were predicted from the genome using Prodigal version 2.50 (6), which revealed 274 open reading frames (ORFs). The proteins encoded by these ORFs were functionally annotated using BLAST searches (http://blast.ncbi.nlm.nih.gov) against the GenBank and UniProt databases.

The UFV-AREG1 genome comprises 170,788 bp of a linear dsDNA with a density of 1.60 gene/Kb and a G+C content of 35.3%, which is within the range observed in T4-like phages (35.0% to 43.0%). UFV-AREG1 is more similar to *Escherichia* phages HY01 and PEC4 (KR233165), sharing 91.8% and 91.5% nucleotide sequence identities, respectively. The 274 predicted proteins show high similarity to previously described enteric phages and other T4-like phages. As a T4-like phage, the genome of UFV-AREG1 was also opened to the *rIIa* gene. The BLASTx searches showed that the genome of UFV-AREG1 differs from the *Enterobacteria* T4 (NC_000866) phage in 30 ORFs. Most of the proteins encoded by these ORFs do not have putative conserved domains. Among these proteins, four annotated ones in UFV-AREG1 related to the T4 phage were found: one receptor recognition protein (ORF 244), one transferase protein (ORF 42), and two structural proteins (ORFs 29 and 121).

The best understanding of the gene and proteins encoded by the UFV-AREG1 bacteriophage can provide new information about *E. coli* phages and their genetic diversity.

### Accession number(s).

The genome sequence of UFV-AREG1 is available in GenBank under the accession number KX009778.
